# Local Positioning Systems in (Game) Sports

**DOI:** 10.3390/s111009778

**Published:** 2011-10-19

**Authors:** Roland Leser, Arnold Baca, Georg Ogris

**Affiliations:** 1 Department of Biomechanics, Kinesiology and Applied Computer Science, Centre for Sport Science and University Sports, University of Vienna, 1150 Vienna, Austria; E-Mails: arnold.baca@univie.ac.at (A.B.); ogris@spantec.at (G.O.); 2 Spantec GmbH, Gumpendorferstraße 132/2/5, 1060 Vienna, Austria

**Keywords:** intelligent space, position measurement, game sports

## Abstract

Position data of players and athletes are widely used in sports performance analysis for measuring the amounts of physical activities as well as for tactical assessments in game sports. However, positioning sensing systems are applied in sports as tools to gain objective information of sports behavior rather than as components of intelligent spaces (IS). The paper outlines the idea of IS for the sports context with special focus to game sports and how intelligent sports feedback systems can benefit from IS. Henceforth, the most common location sensing techniques used in sports and their practical application are reviewed, as location is among the most important enabling techniques for IS. Furthermore, the article exemplifies the idea of IS in sports on two applications.

## 1. Introduction

About 10 to 15 years ago only top-level teams utilized computer assisted video annotation systems in order to analyze game sports and training sessions. Due to the ensuing progress in hard- and software development, nowadays this technique is occasionally even used at the amateur level. However, much more sophisticated technical solutions are now applied in the top-level domain. Vision based position tracking systems have been developed in order to analyze tactical behavior or to obtain information on physical loads; GPS-systems are applied to analyze training sessions of outdoor sports. Radio wave based tracking systems have proven to be a more accurate alternative under training conditions, for both indoors and outdoors. Up till now the potential of identifying player positions in game sports in almost real time has not been utilized in intelligent spaces (IS). Within this paper, considerations are made on how IS, based on position sensing technologies, could be designed for game sports in order to assist players and coaches in their activities. Starting point will be the concept underlying IS, which envisions applications utilizing state-of-the-art information and communication technology (ICT) enhanced by the concepts of pervasive, ubiquitous and contextual computing in order to:

“*improve our abilities to sense and make sense of the physical world about us, and use this knowledge to augment our capabilities as we deal with this world, or to augment our experience of it.*” [1]

Hence the goal of IS applications is to:

“*accomplish an environment that comprehends human intentions and satisfies them.*” [2]

It provides:

“*a space [...] which has ubiquitous distributed sensory intelligence [...] and actuators [...] to manipulate the space [...].*” [2]

Thereby an intelligent space is supposed to sense and act in an unobtrusive way, applies high security and privacy standards, utilizes low power and dynamic communication technologies and provides universal connectivity and high interoperability in order to be open to third party products.

IS in (game) sports was not discussed so far. Anyway, the fact that a certain intelligent sports feedback system may be seen as an IS in the sense of the definitions given above does not *per se* result in a usable feedback system that benefits the users. Nevertheless, the authors state that IS systems can be seen as role model for intelligent sports feedback systems, *i.e.*, when implementing such a system the concepts of IS may guide the actual development process. For the purpose of applying this concept to sports (and game sports in particular) a description of those intentions and needs is required (see also Section 1.1). Moreover, it has to be considered

- which of these intentions and needs can be captured in real-time by state-of-the-art technologies,
- the prediction/satisfaction of which intention/need may add a value for either the sportsmen (professional or non-professional), their coaches and sponsors or the audience.

### 1.1. Informational Needs in Game Sports

Table 1 states a number of examples related to game sports and possible enhancements gained through the assistance by IS (note that the items in the list are not based on each other and that it is not an exhaustive listing of all possible objects!). First of all, the coaches’ intentions and needs can be distinguished between physiological factors and tactical factors. Amongst the physiological factors the main interest concerns the issue of fatigue and its emergence during game play and training. Parameters describing the decline of running performance, for example, can provide the coach with essential information for the decisions of substitutions. For tactical analyses during game play coaches need above all descriptive feedback about several tactical elements. With this information they can assess the efficiency of their tactical behavior and decide if tactical changes are necessary (e.g., switching playing positions or changing the playing system). Furthermore, Table 1 contains some mixed parameters, which are a combination between the mentioned physiological and tactical factors. These variables as well can serve for decisions according to substitutions, playing position shifts and tactical orders to the players during the game.

As can be seen in Column 3 of Table 1 all the scenarios rely—amongst other factors—on positioning data and location information.

### 1.2. Outline

Building on the considerations made in Section 1.1, Section 2 reviews how position measurements are currently utilized in game sports, while Section 3 outlines the technological basis for the underlying position measurement systems. Furthermore, Section 4 exemplifies the concept of IS in game sports on two applications and Section 5 summarizes the review and concludes the main findings.

## 2. Position Measurement in Sports

The use of position sensing technologies is widespread in (applied) sports science research. Since the results of this research have an impact on the development of training programs in practice, it seems important to discuss and get a good overview on applications in this area by reviewing contemporary literature on targeting sports (game sports) and positioning measurement systems.

For such purposes a systematic search of ISI-Web of Knowledge and PubMed using the following keywords was done: position measurement sport; spatial variables measurement sport; intelligent space sport; time-motion analysis sport; sport monitoring position; movement pattern sport; GPS sport; UWB sport; LPM; location monitoring sport; location monitoring performance; location information sport. In particular cases several other search sources were used occasionally when the findings in ISI-Web and PubMed resulted in too small numbers of contributions. The focus of interest of this literature survey is on athletes (real human beings) performing game sports activities. Therefore, search results in the domains of Robo Cup, Virtual Reality and mere Motion Capture were not taken into account.

The published articles in the above described research field can be categorized in various areas. A number of articles were found focusing on descriptions of positioning systems and accuracy measurement. This issue is outlined in Section 3. Almost all other papers deal with time-motion analysis (TMA). The term “time-motion analysis” embraces studies addressing the physiological strains in sports, the development of (playing position specific) player profiles, the analysis of match running demands and work rate profiles. For these investigations manual tracking, (semi-) automatic vision based tracking and GPS-tracking methods are used in the published literature. Articles using manual tracking features were only considered for the review if there was any other position sensing hardware involved.

TMA research can be subdivided in the following fields [3]: analysis of data on overall work rate, categories of movements, determining positional demands, use of motion analysis in studies of fatigue and other uses of motion analysis. Table 2 gives an overview on the absolute and relative frequencies of TMA literature concerning this allocation. Since articles mostly belong to more than one category the summative number of percentages is higher than 100.

Almost all studies addressing overall work rate analysis distinguish between different categories of movements [4–42]. In nearly all cases the overall work load is expressed as distance covered in a game or during specific periods of a game (e.g., first/second half). The categories of movement are typically expressed in dependency of the intensities (speeds) of measured motions and can be categorized in standing, walking, jogging, cruising (striding) and sprinting [3]. Moreover, specific questions are examined in a couple of investigations and additional performance measures are taken. Several studies investigate the demands of sports in correlation to the physical load of individuals: Heart rate [8,13,18,23,36,37,43], lactate [14,18,23,43], oxygen uptake [43] and rating of perceived exertion [23] are measured to assess various metabolic and functional responses of game players. Specific interests in sport science also concern differences of physiological performances between different skill levels [22,27] or different national leagues [15,19,35], between different formats of game play [23,25,26,34,36,37], between the training and game play [8,44] and in the relationship between match performance and the results of diagnostic tests [12,45]. Many of the above mentioned studies compare also the positional demands of game sports [4,6–8,11,14–17,21,28–30,32,35,38,40,42,46] since the kind and intensity of activities in game sports differ from playing position to playing position. Embedded in overall work rate analyses, several articles also address the issue of fatigue in game play [5,9,11,15,18,19,32,33,35,36]. The main aspect therein is the change (decrease) of performance parameters like covered distances or running speeds over the progress of time in competition.

There are few references in TMA research examining other than the above-mentioned issues: The presentation of TMA results to experts enables the opportunity to identify relevant aspects of specific sports for performing team and player related game analysis [47]. In soccer, two studies concerning running path changes in match play were found [48,49], one of which also analyzes the issue of injury risk. Another investigation deals with effects of video-based perceptual training on pattern recognition and pattern prediction ability in elite field sport athletes. The study determines whether enhanced perceptual skills influenced the physiological demands of game-based activities [50]. Investigations addressing the analysis of tactical behavior in game sports by means of position data are very rare. Although the component of tactics has a similar impact on the overall game performance than the physical capacity of players, there are only a few authors devoted to this topic [51,52].

The high number of studies applying manual notation or video annotation for the purposes of TMA (e.g., [53–59]) proves that the results of this kind of research are important for contemporary sports science. Advanced position sensing techniques (see Section 3) nowadays ensure a significantly less amount of work to gain the necessary position data. Referring to the results of the review above, the most important topics in sports science investigating position data in game sports are workload analyses in training and above all in competition as well as evaluations of physiological responses to workloads measured by heart rate, lactate and other metabolic parameters. Furthermore, there are approaches to use game analyses results as an alternative method to diagnostic measurements with the main interest in the issue of fatigue. In addition, differences depending on the playing positions are also considered in most of the mentioned aspects.

The main benefit of the investigation results is in the approved knowledge for designing fitness programs for specific game sports, positional roles and individual players. Moreover, the physical preparation for competitions and development of playing strategies can be done more purposefully. There is one major aspect that is not considered in all of the mentioned research: There was no need of real time analysis in any of the studies. Providing information in real time may be a key aspect in IS for practical sports applications. A series of additional features like immediate feedback or advice (see Table 1) can be done through spatial real-time analysis.

## 3. Position Sensor Technologies

As argued in Section 1.1 the utmost important technology enabling IS in (game) sports is a proper location sensing technique suitable for the specific application in mind. For many of the applications sketched in Table 1 the location information can serve as a basic source of information for analysis beyond mere calculus of covered distances or the like and thus need to have a high enough sampling rate and spatial resolution, and a sufficient spatial accuracy—depending on the specific demands of the sport of interest. Moreover, the technology must be suitable, non-disruptive and legal in terms of the rules of the specific sport.

Location sensing technologies can be split into several categories (For a more detailed categorization of localization systems and position measurement techniques refer also to [60]):

- Target setting: outdoors *vs.* indoors *vs.* mixed
- Basic technology: vision based *vs.* TOF (time-of-flight)/TDOA (time-difference-of-arrival)/AOA (angle-of-arrival)/FDOA (frequency-difference-of-arrival) measurements *vs.* dead reckoning *vs.* hybrid systems (Location systems may also rely on other physical principles. The technologies listed here are those mostly relevant for sports applications.)
- Degree of automation: automatic *vs.* semi-automatic
- Multi-modality: multi-agent *vs.* single-agent *vs.* sequential
- Spatial resolution: centimeters *vs.* meters *vs.* room-level *vs.* location class
- Room centered *vs.* user centered (tracking *vs.* positioning *vs.* location)

In addition to these categories the available technologies differ in terms of sampling rate, spatial resolution, accuracy, the grade of the dependency of the accuracy on current conditions, e.g., position of the athlete, instantaneous dynamics of the athlete and so forth.

Location sensing based on distance measurements [61] is state-of-the-art in tracking and navigation applications [62] especially in indoor applications. Indoor robot and user tracking, positioning and navigation were one of the first research areas for such location sensing techniques. Due to its simplicity and low demands to processing power and synchronization as well its high accurate distance measurements (down to 0.01 m accuracy under optimal and static conditions) ultrasound was one of the first technologies in order to provide such distance readings and thus position estimations [63–65]. The actual implementations vary, but in general these systems relay TOF measurements between base stations with known positions and moving tags. In general ultrasonic based positioning has got several drawbacks. It is in particular subject to reflections and occlusions. Ultrasonic distance measurements essentially require line-of-sight between the communicating devices. In case the transmitter turns away from the receiver or some person/object comes between the two, the signal is lost or a redirected (and thus false) signal is received. Secondly, due to the speed of sound (about 340 ms^−1^) its highest possible sampling rate is quite low compared to human motions. The average achievable sampling rates are between 1 to 5 Hz, depending on the actual system design, implementation and setup.

Moreover, the system has to operate in consecutive mode, *i.e.*, specific distance measurements have to be conducted separately. In the so called user-centered mode (fixed base stations are sending consecutively ultrasonic chirps while mobile tags are operated as receivers and are listening to these signals) the system design scales for an infinite number of moving agents. At the same time the distance measurements to the base stations are not simultaneous which dramatically decreases the position accuracy. Even more, the accuracy highly decreases with the instantaneous speed and dynamics of the athlete. In case that the signal strength is designed for distance measurements of up to 10 m and temperatures down to −10° Celsius, then the time slot for each distance measurement must be at least 0.031 s (not considering an additional time-slot for synchronization and processing) and thus the athlete could be covering a distance of up to 0.31 m in between two consecutive distance measurements. Such an inaccuracy is not viable for most IS applications in sports as described in Section 2. In case the ultrasonic system is operated in the so-called room-centered approach, *i.e.*, each mobile agent is sending an ultrasonic chirp while the fixed base stations are operated as receivers, the number of mobile devices is limited or rather the sampling rate is limited by the number of mobile devices and thus by the number of athletes. In case of 22 athletes (as would be the case when tracking two football teams) the maximum sampling rate (adopting similar considerations as above) would drop to approximately 1.5 Hz. Depending on the dynamics of the specific sport this could be just enough. For high dynamic team sports like football this update rate is too low for many applications.

Paper [66] investigated a broadband ultrasonic system that outperforms the widely used narrow-band ultrasonic positioning systems significantly, though it also increases the complexity of the sensor system. The presented broadband approach enhances the above-mentioned narrow-band ultrasonic systems by means of eliminated interference problems and increased update rates and thus low latency positioning. Anyhow, there is yet no ready-to-use system based on this technology available.

As a successor of ultrasonic positioning systems ultra-wide-band systems emerged. These systems (e.g., Ubisense RTLS, Ubisense, Cambridge, UK) are based on TDOA and/or AOA measurements of ultra-wide-band radio signals between mobile tags and fixed base stations. Due to the faster signal speed much higher sampling rates (approximately 135 Hz for Ubisense) than with ultrasonic positioning systems are possible at an accuracy of down to 0.2 m, *i.e.*, approximately 20 times higher as the maximal achievable accuracy of an ultrasonic positioning system. In multi-agent mode the mobile devices share the overall sampling rate but still the sampling rate is higher than with ultrasonic-based solutions.

Another radio-signal-based solution called LPM presented by Abatec AG (Regau, Austria) [67,68] relies on the distance measurements between fixed base stations and mobile tags based on the frequency modulated continuous wave principle. The system has an overall sampling rate of 1 kHz (again shared amongst the operated mobile tags) and a static accuracy of approximately 0.1 m. Thus, it outperforms Ubisense by a factor of 2 in terms of accuracy and approximately by a factor of 7 in terms of sampling rate. A football game e.g., could be recorded at approximately 45 Hz per player.

Systems that rely on distance measurements (or pseudo-distance measurements as is the case with Ubisense RTLS and Abatec LPM) between base stations and mobile devices also differ in terms of the complexity of deployment, *i.e.*, on-site installation of the fixed stations, calibration of the setup and the scenery, software configurations (e.g., definition of the scenery, the mobile agents and so forth). Depending on the system this process can be rather complex and time-consuming or rather automated and straight forward.

A major drawback of systems demanding the subject of interest wearing a tag is the fact that for ball game sports the question how the ball can be tracked stays an open issue so far. Attempts to integrate a tag into the ball itself are just a work-around applicable for proofs of concept but not for ready-to-use systems. This is mainly due to the fact that the ball's characteristics are altered which is in no way acceptable—especially when it comes to professional sports (On the other side, the Goal Line Technology [69] proves that ball tagging is possible.). What is more, tags are simply not allowed during competition matches though for training sessions and most athletes and teams refuse to wear such sensors or tags arguing understandably that the players’ performance is disturbed and the risk of injuries increases.

In team sports (semi-automatic) video tracking (see e.g., [70–73]) of the athletes currently prevails. Impire AG (Ismaning, Germany) and Cairos Technologies AG (Karlsbad, Germany) e.g., provide a ready to use vision based tracking system for team sports called VIS.TRACK. The system consists of two cameras and tracking software in order to track both the players and the ball. The major advantages of vision based systems are their high update rate (corresponding to the camera frame rate) and the fact that the players and the ball are tracked simultaneously, *i.e.*, each position sample for a single player has got a corresponding position sample for every other player (including the ball) measured at the identical point in time. The major drawback of such systems is their semi-automatic tracking algorithm. Operators have to perform player identification at the beginning of the game and thereafter throughout the entire play as soon as the tracking software mixes two or more players—which is likely to happen due to crowded situations, e.g., during an offense situation within the goal area, a corner play, goal cheer and so forth. Furthermore, the accuracy of vision based systems decreases with increasing distances between players and cameras. Paper [74] investigated this deficiency on football pitches.

VIS.TRACK overcomes these drawbacks by providing additional information to the vision based tracking system via GPS tags worn by each player. These tags are operated at 10 Hz each. This hybrid vision-GPS-based tracking system is thus capable of providing player position information in a fully automatic manner. Another solution to the automatic person identification for video based tracking was presented in [75]. The authors introduce a hybrid tracking method based on computer vision in order to track persons passing through the video surveyed area but also to perform a rough activity classification of the tracked persons. This classification result is then matched with body mounted acceleration based activity recognition in order to perform automated user identification. A remaining drawback of such hybrid systems is the fact that the ball tracking still cannot be fully automated.

A major advantage of the systems described so far is the fact that they provide a good enough spatial accuracy (though sampling rates may be too low) for many of the applications discussed in Table 1. Secondly, the accuracy and sampling rate does not alter over time and what is more, the coordinate systems for both systems can be linked during the deployment stage—more exactly during the calibration stage—with the navigation frame, *i.e.*, the sports field and does not change over time as well. These advantages are not inherent in every location technique, as is the case for, e.g., INS/GPS navigation [76].

In case of outdoor scenarios GPS based positioning [77,78] is of course a widespread approach, mainly because its deployment is quite easy. Various studies (see also Section 2) and applications (e.g., [79]) attest GPS its applicability for spatiotemporal sports analysis. Nevertheless, the accuracy of GPS is dependent on the GPS receiver, the actual GPS satellite configuration and on whether an enhancement technology is applied, e.g., Assisted GPS (AGPS) or Differential GPS (DGPS). In general, GPS is less reliable concerning its accuracy, specifically because the accuracy is very unstable. Secondly, update rates may be too low for certain applications and still it relies on a tag being worn on-body. Tag-related shortcomings were already discussed earlier in this section. On the other side its advantages over other location techniques are quite obvious:

- Players can be tracked independently, *i.e.*, non-consecutively (note, that the tracking of the tags is non-consecutive, but the measurements are not done at identical points in time either.)
- Constant up-date rates per tag that do not have to be shared amongst other tags.
- Easy and fast deployment: GPS services are available outdoors almost everywhere.

Advances in the so-called pedestrian dead reckoning (PDR) [80–82] approach to the problem of indoor positioning let assume its applicability also for IS in sports applications. PDR estimates the position of the user by means of integrating acceleration, velocity, rate-of-turn measurements or a combination of these—often referred to as inertial navigation system (INS). Due to the fact that the thus derived position estimations are subject to drift over time, such position systems usually rely on aiding sources, *i.e.*, measurements that provide absolute reference data. The aiding source can be, e.g., an absolute position measurement provided by GPS. Such hybrid INS/GPS positioning systems combine the advantages of both systems: drift-less absolute position measurements by means of the GPS measurements with the high update rates (approximately 100 Hz) of the inertial navigation system.

Summarizing this short overview on location sensing technologies currently applicable to sports, we state that none of these solutions is fully meeting the requirements of IS so far. State-of-the-art technologies come with at least one of the following deficiencies: they lack an unobtrusive character; are maybe not easily deployed; have a too low accuracy and/or provide an unacceptable update rate. Whether or not these deficiencies are relevant depends on the field of applications. Nevertheless, current technologies provide a good basis for prove of concept experiments and setups.

## 4. IS in Game Sports

There have been sports related developments, which reveal characteristics of IS and which rely on location sensing technologies. GPS-based devices in running, for example, provide target-performance comparison and give feedback on covered distance and speed. Information on advantage or deficit is given in order to enable athletes to adjust their speed accordingly. In [83] a system is proposed, which automatically selects between several tracks in a cross-country running in order to keep the expected heart rate in a target interval. Integrating position data and 3D-maps of the training area might extend the concept. Runners (or mountain bikers, cross country skiers, *etc.*) might thus get advice, on where to continue their run (ride, *etc.*) depending on their actual position. Paper [84] introduces a prototype system for controlling the training of a group of cyclists. The cyclists automatically get instructions on how to change their current formation according to their physical strain. However, to our knowledge, no such developments have been reported for game sports. The idea of IS shall therefore be exemplified on two applications based on location sensors.

### 4.1. Example I: Mobile Coaching

The Mobile Coaching system has been developed to evaluate performance parameters during training or competition and to provide athletes with feedback. Physical loads of athletes may consequently be adapted with respect to their individual performance level [85,86], recommendations on how to continue the sports/physical activity are given. Parameters characterizing the performances of a group of athletes may be monitored and observed continuously. Thus, coaches are able to supervise and support the athletes individually and in parallel. The athletes get feedback on the quality of their motion/activity utilizing sport scientific knowledge. In addition, their performances may be documented.

**Figure 1.**
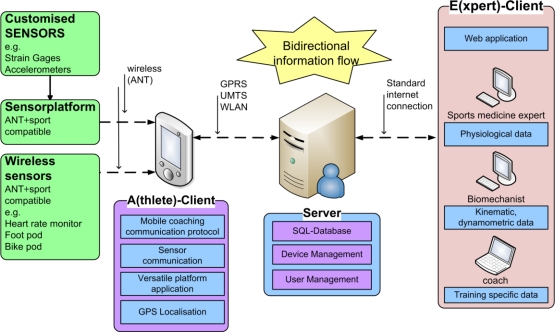
Overview of the Mobile Coaching System (*cf.* [86]).

In order to obtain performance parameter values like heart rate, velocity or reactive forces, sensors are either attached to the athletes or mounted onto their sports equipment. The measured data is transmitted to a smartphone via a wireless sensor network (WSN). From there the data is sent to an application server via Internet. Feedback is either generated automatically by a server application or individually provided by the coach (or an expert having web access to the server). A system overview of the MMA is shown in Figure 1.

The current implementation of the system integrates wireless sensors based on the ANT+ protocol, which is well established for practical applications in the area of sport. The Mobile Client(s) (A-client; see Figure 1) hardware utilized by the athlete(s) includes a smartphone with ANT+ connectivity (e.g., Sony Ericsson Xperia Arc, Android). The client application software is implemented in Java.

The A-client application is listening on different channels for incoming signals from surrounding sensors. In addition, the current GPS location of the athlete is identified. The measurement data is saved temporarily and—in case the mobile device is connected to the Internet—immediately sent to the host component, where it is stored in an SQL database.

The E-client utilized by the coach (or expert) is realized using PHP and JavaScript and runs on an Apache™ HTTP Server. The recently processed sensor data like the number of strides, distance and speed or current heart rate are displayed. A tool for visualizing the progress of the measured parameters (for example the heart rate values) in real time is available. In this way, coaches (experts) are able to observe/analyze the athletes’ performance throughout the entire training/competition. A specific feature allows them to return individual feedback messages. Such notifications are sent back to the mobile device via the server. They are displayed as a text message including an alert beep and vibration signal. In addition, feedback messages optionally may be given in form of voice output over the integrated loudspeaker.

Table 3 gives a survey on some parameter/sensor combinations in selected sports including game sports. Considering game sports in more detail, the question arises on how to integrate position data, which do not originate from GPS receivers, into the system. As has been outlined in Section 3, most other technologies do not provide the position data at the device carried by the user. Anyway, it should be possible to transfer the position data to the server, where they may be combined and synchronized with the physiological, biomechanical, etc. athlete data.

Assuming that there are appropriate software components available on the server, which are capable of analyzing and rating tactical behavior, intelligent feedback could be provided for coaches and athletes. Currently, some ongoing developments in this area can be observed. Methods for tactical pattern recognition based on self-organizing maps are presented in [51] and [52]. In future, coaches might thus get advices during the match on how to give direction to tactical behavior. Athletes might receive feedback information during game breaks. This would particularly be viable in training matches.

On the other hand, models could be applied for predicting strain responses due to physical loads as can be estimated from running intensities and distances. Such models are presented in [87] and [88] and are currently evaluated in combination with the Mobile Coaching system in endurance sports. Coaches and athletes could thus be provided with respective information during the match and substitutions could be planned in time, if necessary.

Despite the fact that the most essential MMA-features characterizing an IS (see above) are currently still under development, the mobile coaching system might act as universal platform for intelligent systems in sports in future. The system provides interfaces for a variety of sensors, which are able to measure sports performances, as well as the infrastructure to process this data with intelligent methods and to return the results of the processing to the target audience near to real time.

### 4.2. Example II: Game Analysis

Computer-assisted game analyses support coaches to prepare their teams for coming opponents and to evaluate the game behavior of the own team [89]. Analysis systems provide post match video data and statistics to assess the performance of teams and individual players. Table 1 presents several parameters coaches are interested in. Considering the concept of IS (Introduction), this kind of parameters should especially be supplied in real time. At present, no game analysis systems are available with this capacity but a few approaches are reported. One of the most sophisticated concepts in this field is the soccer interaction and process model introduced in [90]. The computerized real-time analysis system for soccer games acquires action models, infers action-selection criteria and identifies player as well as team strengths and weaknesses using a positioning system. The system's components are explained in detail in [91]: The model is grounded on a vision based observation system, which provides position data and trajectories. Estimating several camera parameters, such as the position of the camera related to the field as well as the pointing direction and the zoom factor from a couple of synchronously used cameras, even broadcast video streams can be used to continuously calibrate the observation field and to provide position data from players and the ball in real time.

**Figure 2.**
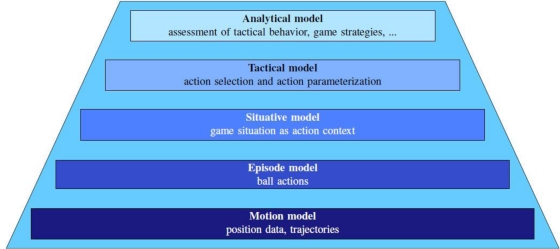
Hierarchical structured models for analyzing soccer games based on position data ([91], with permission).

The second main component of the system is a hierarchically structured model for soccer games (Figure 2). The position data and trajectories from the observation system are coincidentally the lowest layer of the model. Each model layer builds concepts for the next higher level. On the basis of the motion model (position data and trajectories) several evaluations on physical activities like covered distances and intensities can be done. On the next higher level (episode model) ball actions are identified by means of the position data and trajectories, which in turn help to identify game situations on the upper model layer (situative model). If game situations like special types of attacks or defensive behavior of teams and players can be determined, it is possible to assign the tactical behavior of game sports actors in a next step (tactical model). The last layer on the top of the system hierarchy is the analytical model. This model provides routines to assess the quality of actions and situations and to rate tactical behavior in the context of the game. Furthermore, game strategies can be detected here with the help of the tactical behavior determined in the lower level models before.

The automated sports game models already provide exemplary analysis results [91] especially for the episode and the situative model. For more sophisticated information, in particular for the determination and assessments of tactical behavior in its game context and for game-strategic analyses, improved methods of position data processing are necessary at the high level layers. In [91] statistical learning methods are proposed for this. Other approaches dealing with neural networks were already mentioned in Section 4.1 [51,52].

An example to illustrate the game analysis model as an application of IS in game sports can be considered by the issue of pressing. Pressing is a basic principle in game play, where the defensive team tries to gain ball possession by systematically covering field zones and opponent players [92]. Although there are very general rules for playing pressing effective, each team has an individual style to perform it. Knowing those details of an opponent team can help to advance the preparation of the own team and above all, to improve strategic decisions during competitions. Two main factors of individual pressing styles are the location (where on the field) the player in ball possession is attacked (offensive third *vs.* midfield third) and if the collective pressing action is started through a key event (e.g., time instant, when the ball is played to a wing player). Furthermore, it is advantageous to know for the offensive team, if the opponent team has any systematic shortcomings in their defensive behavior (e.g., zones they often don’t cover optimally and which can be used for attacking). Having the position data and trajectories of players in pressing situations the mentioned issues could be analyzed by means of AI-methods [93,94] and the coach could receive automatic feedback for tactical instructions during the game.

The game analysis concept described above seems to be a prime example for an IS application in game sports. Parameters calculated on the basis of the position data can be used during the competition to assess the physical strain of the players. Results from the episode and the situative model give a good overview of performed actions and observed situations in real time. If working fully functional, the high level layers of the model can assist the coach in tactical and strategic decision making. This coaching need is without a doubt a key factor for game analyses working as IS. Due to the high complexity of game sports and the complex processes of modeling decision making in coaching, there exist partial solutions only for these tasks so far. Few works are reported considering this main problem in its full range.

## 5. Summary and Conclusions

In summary, we state that IS systems could provide information, insights and perspectives for the day-to-day work of coaches and players and thereby ensure high unobtrusiveness and usability. This information has to meet the needs and intentions of the actors and thus should assist them in their practical work. Technological driven developments often suffer from the drawback that they rather provide information that is technological viable than information that is asked by the target group. Therefore, a starting point to realize a concrete IS scenario in game sports could be a listing of needs and arguments similar to those in Table 1. Secondly, the mode of presentation of the information has to be defined. In general, sports men need straightforward statistics that are expressive and definite. Besides mere descriptive information a particular challenge for IS systems is to provide advice for the users. In game sports this could be on the whole, tactical advices to coaches and players, feedback on fatigue and hints for substitutions. For this features the property of real-time capability is very crucial.

Contemporary position sensing technology used for time motion analysis in sports is already very advanced. Anyway, there are still some obstacles to use this technology also for IS. First of all, sensors have to be unobtrusive, meaning that they must not change the sports environment essentially and must not disturb athletes in their activities. Active systems like GPS mostly do not accomplish this requirement because athletes have to wear hardware devices in order to sense their positions and speed. It can be expected that future developments in sensor miniaturization will lead to much smaller positioning devices which can be integrated in the sports equipment (e.g., sewn into textiles) and thus will be less intrusive. Vision based systems are absolutely unobtrusive but have the drawback of only working in a semi-automatic manner at present. This is in particular inefficient during training sessions as no operators are available at sports clubs for this task.

Besides the size of the sensing technique, also the sample rate and the accuracy of the position estimations are crucial factors. Considering the applications in TMA an accuracy of about 15–20 cm under dynamic conditions and a sample rate of at least 10–15 Hz seem sufficient for most game sports. Many of the currently available position systems already comply with these requirements.

Reflecting the most important key points for realizing IS in game sports, we conclude that the present framework conditions for providing proper raw data for IS applications are good. Existing obstacles can be expected to be solved through technological development in a few years. What remains is a lack of methods to process this raw data for concrete IS applications so that this information can be used purposefully. Up till now, only few promising approaches (e.g., [51,52] for tactical pattern recognition) have been reported. Nevertheless, there exist attempts which deal with as complex issues as creativity learning—a crucial capability in tactical performance analysis—in game sports by now [95]. Therefore, it is expected that this current key problem of IS applications in game sports can be solved.

## Figures and Tables

**Table 1.** Typical game sport specific demands and solutions according to IS.

**Intentions/Needs**	**Satisfactions/Variables**	**Technology**
**Analysis of physiological factors**		
Assessment of player's fatigue during match play Recognition of physical overload of the players in due time Feedback on exertion of players in training	Overall running performance of individual players (distance in meters) Running performance according to different intensity levels (walking, cruising, running, sprinting) Maximum speed of individual players (km/h) Overall running performance of the team according to the time interval (1–15 min, 16–30 min *etc*.)	High-resolution position and speed measurements (HRPS) Modeling and recognition of the player’s physical context (MRPPC)

**Analysis of technical-tactical behavior**		

Feedback on the observance of playing strategies (team, groups of players, individual players) Recognition of the opponent’s match play strategy Recognition of tactical strengths and weaknesses in the playing behavior Information on tactical behavior for spectators	Number of passes Ratio between different types of passes (short/wide, low/high) Ratio between good and bad passes Number of passing stations per ball possession Number of 1 *vs.* 1 situations Ratio between won and lost 1 *vs.* situations Number of passes into the penalty box Number of shots on the goal, into the goal and beside the goal Ratio of ball possessions in different playing zones (defense, midfield, offense)	HRPS Modeling and recognition of game tactics based on multi-modal positions and trajectories (MRGT) Modeling and recognition of the game context (MRGC)

**Analysis of mixed factors**		

Knowledge of workload in dependency of playing positions, of states of the game and of the course of the game Knowledge of the ratio of workload between different playing positions, states of the game and in the course of the game	Overall running performance of individual players (distance in meters) according to their playing positions Running performance according to different intensity levels (walking, cruising, running, sprinting) and playing positions Overall running performance of the team according to the current score Overall running performance of the team according to the percentage of ball possession Overall running performance of the team in dependency of the tactical playing concept in defense and offense	HRPS MRGT MRGC

**Table 2.** Allocation of time-motion analysis literature.

	**Frequency**	**Percentage**
Analysis of data on overall work rate	45	82
Analysis on categories of movements	45	82
Analysis of positional demands	21	38
Use of motion analysis in studies of fatigue	10	18
Other uses of motion analysis	5	9

**Table 3.** Parameter/sensor combinations in selected sports (*cf.* [86]).

**Sport**	**Sensor**	**Parameter**
**Running**	Stride Sensor	Distance, cadence, velocity

**Running/Cycling**	Heart rate monitor (HRM)	HR, HRV
	Location sensor (GPS)	Position, velocity

**Cycling/Mountain biking**	Gear position indicator	Gear position, distance per stride ratio
	Inclinometer	Inclination
	Cadence sensor	Pedaling frequency
	Speedometer	Speed, average speed

**Game sports**	Heart rate monitor	HR, HRV
	Location sensor (GPS, *etc.*)	Position, velocity
